# Supplementary material for: PoMeLo: a systematic computational approach to predicting metabolic loss in pathogen genomes
Source: BMC Bioinformatics. 2024 Jan 30;25:49. doi: 10.1186/s12859-024-05640-w (PMC10829301; doi:10.1186/s12859-024-05640-w)
Supplement: Supplementary file 7 — Additional file 7: Treponema Newick output. This output is generated by PoMeLo during the optional phylogenetic portion of the code. This represents the phylogenetic reconstruction in Newick format. This is used by the program to build a tree, and it can be used by the user to build a tree of their own. [file 12859_2024_5640_MOESM7_ESM.docx]

**Additional File 7: Treponema Newick Output**

((((((((Treponema_peruense_2.74:0.3677332365,Treponema_succinifaciens_2.9:0.401377118)100:0.07291438107,Treponema_rectale_2.86:0.9213763592)100:0.2430015791,Treponema_ruminis_2.9:0.662140719)100:0.09746587777,Treponema_sp_nC27_bin.160.fa_2.06:0.6102320885)100:0.1125478886,(Treponema_socranskii_2.81:0.04152379916,Treponema_sp_Marseille-Q4132_2.73:0.04592599499)100:0.5843902928)100:0.07676467483,Treponema_parvum_2.66:0.5998086126)100:0.2080606893,Treponema_brennaborense_3.06:0.5078683375)100:0.2676449572,(((((((Treponema_sp_OMZ_791_3.09:1.0000005e-06,Treponema_sp_OMZ_789_3.07:1.0000005e-06)18:1.0000005e-06,Treponema_sp_OMZ_790_3.09:1.0000005e-06)100:0.08720277311,((Treponema_sp_OMZ_787_2.76:0.00786284049,Treponema_sp_OMZ_788_2.81:0.008535327341)100:0.05951181588,(Treponema_sp_OMZ_799_3.04:0.006524798209,(Treponema_sp_OMZ_792_2.92:1.0000005e-06,Treponema_sp_OMZ_798_2.89:1.0000005e-06)100:0.007541688546)100:0.03634795996)100:0.0182968124)100:0.03202786659,(Treponema_putidum_2.8:0.09620177457,(Treponema_sp_B152_2.76:0.01373371637,Treponema_denticola_2.84:0.01503560291)100:0.0442280496)100:0.03261443935)100:0.2628547984,Treponema_pedis_2.89:0.3195825511)100:0.4094656726,((Treponema_phagedenis_3.37:0.3766215424,(Treponema_pallidum_1.14:0.00452725271,Treponema_paraluiscuniculi_1.13:0.004694900872)100:1.160395538)100:0.1896802461,((Treponema_sp_OMZ_855_2.69:0.01465477395,Treponema_sp_OMZ_857_2.84:0.01719007462)100:0.03274909938,((Treponema_sp_OMZ_803_2.8:0.009770821151,(Treponema_vincentii_2.98:0.008556801208,Treponema_sp_OMZ_906_2.81:0.008527134453)91:0.003530071316)100:0.05842747716,(Treponema_sp_OMZ_305_2.63:0.06300161414,Treponema_medium_2.72:0.04037598389)100:0.01754306623)9:0.01473431182)100:0.5451938547)61:0.08759950223)100:0.3873096379,Treponema_primitia_4.06:1.363728961)100:0.09718220071);

Additional File 7: Treponema Newick Output. This output is generated by PoMeLo during the optional phylogenetic portion of the code. This represents the phylogenetic reconstruction in Newick format. This is used by the program to build a tree, and can be used by the user to build a tree of their own.
