# Supplementary material for: PoMeLo: a systematic computational approach to predicting metabolic loss in pathogen genomes
Source: BMC Bioinformatics. 2024 Jan 30;25:49. doi: 10.1186/s12859-024-05640-w (PMC10829301; doi:10.1186/s12859-024-05640-w)
Supplement: Supplementary file 8 — Additional file 8: Computational time analysis. All times shown in minutes:seconds (m:s) format. [file 12859_2024_5640_MOESM8_ESM.docx]

Additional File 8: Computational Time Analysis.


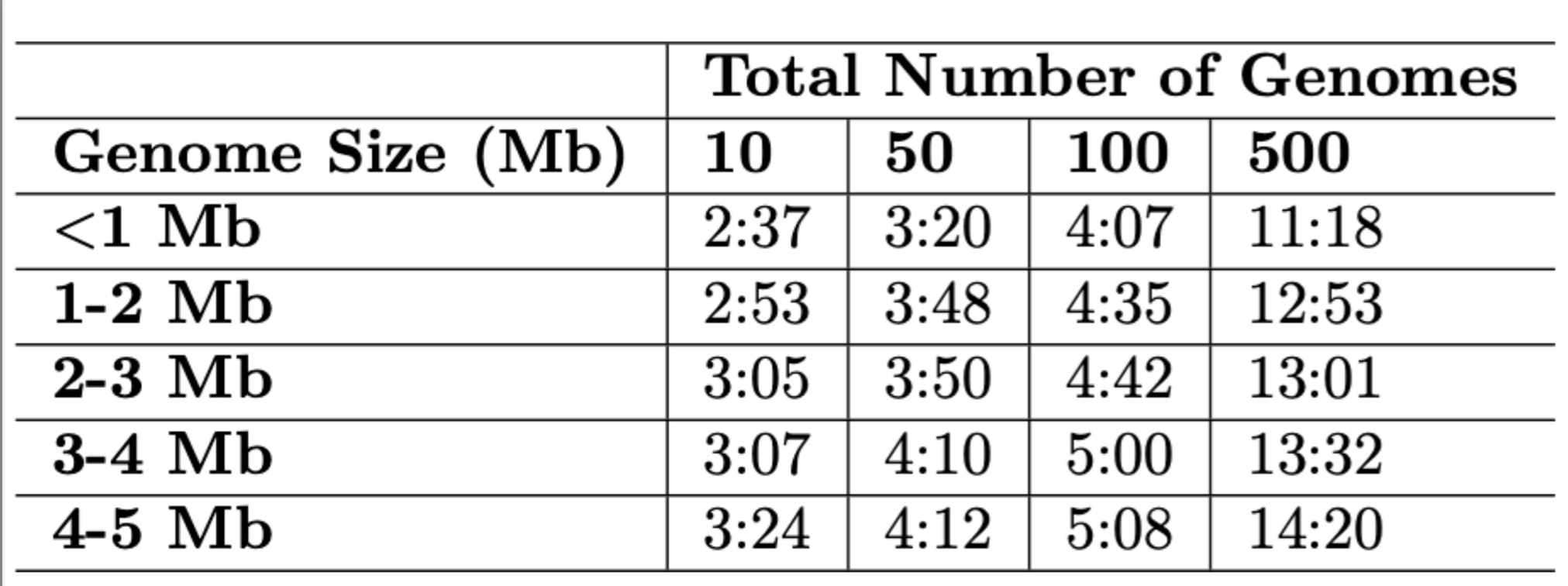


Additional File 8: Computational Time Analysis. All times shown in minutes:seconds (m:s) format.
